# Risk of severe COVID-19 in unvaccinated patients during the period from wild-type to Omicron variant: real-world evidence from Japan

**DOI:** 10.1265/ehpm.23-00274

**Published:** 2024-03-05

**Authors:** Kimiko Tomioka, Kenji Uno, Masahiro Yamada

**Affiliations:** 1 Nara Prefectural Health Research Center, Nara Medical University, Nara, Japan; 2 Chuwa Public Health Center of Nara Prefectural Government, Nara, Japan; 3 Department of Infectious Diseases, Minami-Nara General Medical Center, Nara, Japan

**Keywords:** COVID-19, SARS-CoV-2, Severe outcomes, Wild-type, Omicron, Community-based study, Japan

## Abstract

**Background:**

Many studies have reported that the Omicron variant is less pathogenic than the Delta variant and the wild-type. Epidemiological evidence regarding the risk of severe COVID-19 from the wild-type to the Omicron variant has been lacking.

**Methods:**

Study participants were COVID-19 patients aged 18 and older without previous COVID-19 infection who were notified to the Nara Prefecture Chuwa Public Health Center from January 2020 to March 2023, during the periods from the wild-type to the Omicron variant. The outcome variable was severe COVID-19 (i.e., ICU admission or COVID-19-related death). The explanatory variable was SARS-CoV-2 variant type or the number of COVID-19 vaccinations. Covariates included gender, age, risk factors for aggravation, and the number of general hospital beds per population. The generalized estimating equations of negative binomial regression models were used to estimate the adjusted incidence proportion (AIP) with 95% confidence interval (CI) for severe COVID-19.

**Results:**

Among 77,044 patients included in the analysis, 14,556 (18.9%) were unvaccinated and 520 (0.7%) developed severe COVID-19. Among unvaccinated patients, the risk of severe COVID-19 increased in the Alpha/Delta variants and decreased in the Omicron variant compared to the wild-type (AIP [95% CI] was 1.55 [1.06–2.27] in Alpha/Delta and 0.25 [0.15–0.40] in Omicron), but differed by age. Especially in patients aged ≥80, there was no significant difference in the risk of severe COVID-19 between the wild-type and the Omicron variant (AIP [95% CI] = 0.59 [0.27–1.29]). Regarding the preventive effect of vaccines, among all study participants, the number of vaccinations was significantly associated with the prevention of severe COVID-19, regardless of variant type. After stratified analyses by age, patients aged ≥80 remained a significant association for all variant types. On the other hand, the number of vaccinations had no association in Omicron BA.5 of patients aged 18–64.

**Conclusions:**

Patients aged ≥80 had less reduction in risk of severe COVID-19 during the Omicron variant period, and a greater preventive effect of vaccines against severe COVID-19, compared to younger people. Our findings suggest that booster vaccination is effective and necessary for older people, especially aged ≥80.

**Supplementary information:**

The online version contains supplementary material available at https://doi.org/10.1265/ehpm.23-00274.

## Graphical Abstract

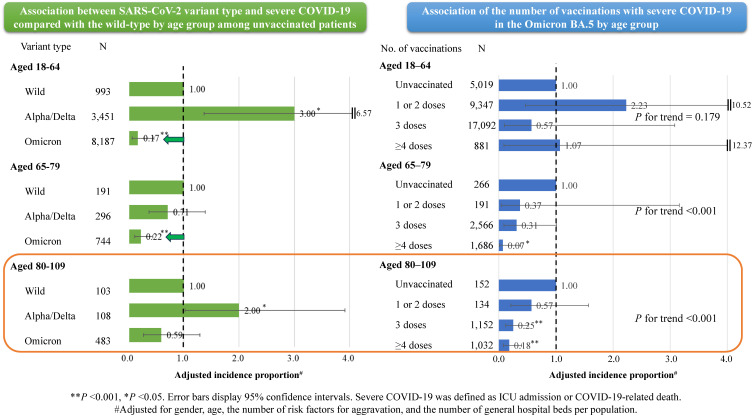

## Background

Coronavirus disease 2019 (COVID-19) makes it difficult to control the spread of infection because there are many asymptomatic infections, the speed of mutation is fast, and breakthrough infection is likely to occur [1, 2]. On the other hand, as the mutation progressed, the proportion of severe cases of infection decreased, so restrictions were eased worldwide. The World Health Organization declared the end of the “public health emergency of international concern” on May 5, 2023 [3].

Vaccination is an important key to combating infectious diseases [4]. Vaccination against COVID-19 is progressing in Japan and around the world. In Japan, advance and priority vaccination for healthcare workers started on February 17, 2021, and priority vaccination for older people started on April 12, 2021. By the end of November 2021, 76.9% of the total population completed 2 doses of vaccination. According to the latest data (as of September 23, 2023) [5], 79.8% of the population have completed the second dose, and 91.5% of older people aged 65 and older have completed the third dose. We have reported that increasing the number of vaccinations can reduce the severity of COVID-19 [6, 7].

It is important to examine whether the reduction in the risk of severe outcomes is due to the weakening of the pathogenicity of COVID-19 or the effect of the vaccine in preventing severe disease. Therefore, we examined the following two points using the data of COVID-19 patients from the wild-type to the Omicron variant reported to a certain public health center. First, with the aim of examining whether the pathogenicity of COVID-19 has been weakened, the risk of severe outcomes in the unvaccinated COVID-19 patients was evaluated from the wild-type to the Omicron variant. By conducting this analysis, we thought that it would be possible to evaluate the pathogenicity of severe acute respiratory syndrome coronavirus 2 (SARS-CoV-2) without being affected by the effect of vaccination to prevent severe disease. Second, we examined the association between the number of vaccinations and the risk of severe outcomes for each variant type, with the aim of examining whether the effect of the COVID-19 vaccine in preventing severe disease differs depending on the variant type. Additionally, because age is a strong predictor of severe outcome [6], age-stratified analyses were performed.

## Methods

### Data and study participants

We used data from the Health Center Real-time Information-sharing System on COVID-19 (HER-SYS) [8]. The HER-SYS is described in detail elsewhere [6]. Briefly, the HER-SYS is an online system that manages information such as the medical history and vaccination history of COVID-19 patients. Doctors who diagnosed COVID-19 were required to submit a notification to the public health center in accordance with the Infectious Diseases Control Law, but by entering information into the HER-SYS, it was assumed that a notification had been submitted. Furthermore, information input into the HER-SYS required not only health status at the time of diagnosis, but also follow-up health outcomes after diagnosis. This study was conducted in the jurisdiction of the Chuwa Public Health Center of the Nara Prefectural Government, which has jurisdiction over 18 municipalities (7 cities, 8 towns and 3 villages) with a total population of 573,438 as of January 1, 2021. At the Chuwa Public Health Center, patient information before the HER-SYS was established was entered into this system after its establishment, and was centrally managed with the HER-SYS. Therefore, we were able to obtain data on all patients after the wild-type from the HER-SYS.

**Fig. 1:**
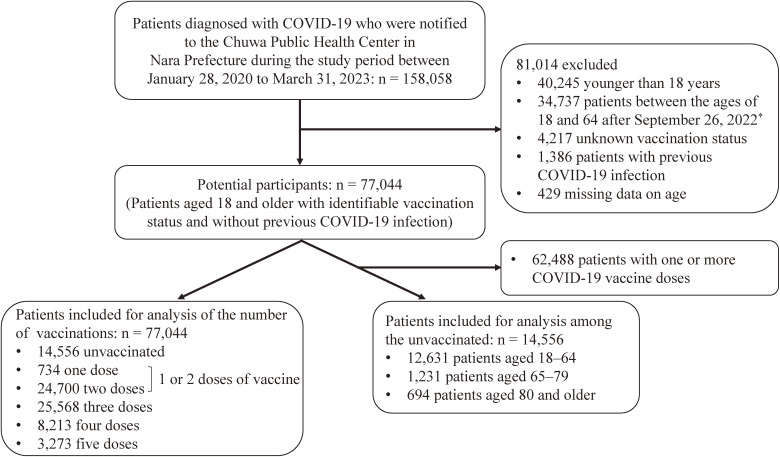
Flowchart of study participants. *After September 26, 2022, people under the age of 64 were exempt from the notification of all cases, and the notifications were limited to those with pre-existing conditions and pregnant women.

Of the 158,058 COVID-19 patients notified to the Chuwa Public Health Center between January 2020 and March 2023, the analysis included 77,044 patients aged 18 and older with identifiable vaccination status and first-time COVID-19 infection (Fig. 1). The reason for excluding patients aged 18 to 64 years after September 26, 2022 is that from this date, notification of COVID-19 patients aged 18 to 64 years was limited to those with risk factors (e.g., patients with underlying medical conditions and pregnant women). In other words, this study was based on patient data during the period when it was possible to capture all patients.

We compared the risk of severe COVID-19 in persons with identified vaccination status and persons with unknown vaccination status (Additional file 1). People with unknown vaccination status were more likely to have severe COVID-19 than people with identified vaccination status, especially among older people aged 65 and older, or during the Omicron variant.

### Period of spread of each variant type

Based on the trend in the number of daily new COVID-19 cases in the jurisdiction of the Chuwa Public Health Center (Additional file 2) and the results of the SARS-CoV-2 genome analysis conducted by the Nara Prefectural Institute of Health (Additional file 3), we defined the period of spread of each variant type as follows: wild-type from January 28, 2020 to February 28, 2021, Alpha variant from March 1 to July 11, 2021, Delta variant from July 12 to December 26, 2021, Omicron BA.1/BA.2 from December 27, 2021 to June 26, 2022, Omicron BA.5 from June 27 to October 10, 2022, and Omicron Mixed period from October 11, 2022 to March 31, 2023.

### Vaccination status

Using the HER-SYS data reported by the doctor, we evaluated the number of vaccinations against COVID-19 on the date of COVID-19 onset. Due to the increase in the number of infected people, the notification was revised three times during the study period. The number of vaccinations was consistently included in the notification. On the other hand, there was a time when the type of vaccine was not included, making it impossible to evaluate the type of vaccine that study participants received. According to a report from the Prime Minister’s Office on the number of vaccinations by prefecture as of September 17, 2023 [5], residents of Nara Prefecture have received a total of 4,209,350 vaccinations, of which 79.2% is by Pfizer, 20.7% by Moderna, and 0.1% by others (AstraZeneca and Novavax). The national average was 77.2% for Pfizer, 22.7% for Moderna, and 0.1% for others. Therefore, Nara Prefecture has a slightly higher proportion of Pfizer than the national average.

### Previous COVID-19 infection status

The HER-SYS, data source in this study, did not include information on previous infection. In Japan, a notification must be submitted to the public health center in the patient’s place of residence. Therefore, for residents who have not moved during the study period, the first notification can be considered as a first infection. In this study, patients who were submitted as a first-time notification were determined to be patients with no previous infection and included in the analysis.

### Health outcome

Health outcome was severe COVID-19. In this study, we adopted follow-up health outcomes after diagnosis as the health outcome; cases who were already deceased at the time of diagnosis were excluded from our analyses. Previous studies have defined severe COVID-19 as hospitalization or death [9, 10]. In Japan, until the fourth wave (i.e., the wild-type and the Alpha variant), all infected people had to be hospitalized regardless of their condition. Therefore, hospitalization during this period is not an indicator of severe disease. In this study, persons with severe COVID-19 were defined as patients with an intensive care unit (ICU) admission or COVID-19-related death.

### Covariates

With reference to previous studies [9–13], we used age, gender, the number of risk factors for aggravation, and the number of general hospital beds per 100,000 population in the municipality where the patient resided (hereafter, the number of general hospital beds per population), as covariates, because they were considered to be important confounding factors in the association between vaccination status and severe COVID-19. Risk factors for aggravation included chronic respiratory disease, chronic kidney disease, diabetes, hypertension, dyslipidemia, cardiovascular disease, cerebrovascular disease, malignant tumors, compromised immune function (organ transplantation, etc.), smoking, obesity with a body mass index of 30 or more, and pregnancy. Using the number of risk factors for aggravation, the participants were classified into three groups: none, one, and two or more. For the number of general hospital beds per population, previous studies have reported that it is associated with the case-fatality ratio of COVID-19 [11, 13]. Using the number of general hospital beds per population, we classified the participants into quartile groups.

### Statistical analysis

We used the generalized estimating equations of negative binomial regression models (which deal with overdispersion) to evaluate the association of SARS-CoV-2 variant type or the number of COVID-19 vaccinations with severe COVID-19 (i.e., ICU admission or COVID-19-related death). We calculated adjusted incidence proportion (AIP) with 95% confidence interval (CI) for severe COVID-19. First, among the unvaccinated participants, we investigated the association between SARS-CoV-2 variant type and severe COVID-19, with the wild-type as the reference. Next, among all study participants, we investigated the association between the number of vaccinations and severe COVID-19 by SARS-CoV-2 variant type, with the unvaccinated group as the reference. This analysis was limited to the Delta and Omicron variants, as general vaccination for people aged 18 years and older began in Japan on June 17, 2021 [5]. These analyses were performed stratified by age group (i.e., aged 18–64, aged 65–79, and aged 80–109).

Using the IBM SPSS Statistics Ver. 27 for Windows (Armonk, New York, US), we performed statistical analyses, with a significance level at 0.05 (two-tailed test).

## Results

Of the 77,044 COVID-19 patients aged 18 and older, the prevalence of people aged 65 and older was 27.7%, the male prevalence was 46.3%, and the prevalence of people with risk factors for aggravation was 37.3%. There were 1,287 (1.7%) wild-type cases, 14,556 (18.9%) unvaccinated individuals, and 520 (0.7%) who developed severe COVID-19.

Regarding characteristics of the study participants by SARS-CoV-2 variant type (Table 1), because we failed to capture patients under the age of 64 in the Omicron Mixed period, data comparisons of proportions between groups were made for the period from the wild-type to the Omicron BA.5. The percentage of individuals aged 64 years or younger was lowest during the wild-type and highest during the Delta variant. Men accounted for the majority from the wild-type to the Delta variant, while women had a majority when the Omicron variant became dominant. For vaccination status, more people received more vaccine doses as the variant progressed. Cumulative incidence of people with severe COVID-19 showed a significant trend in decreasing proportion as the variant progressed (Cochran-Armitage test for trend, *P* < 0.001). Regarding characteristics of the study participants by age group (Additional file 4), with increasing age, there were more women, more people with aggravation risk factors, fewer unvaccinated people, and more severe cases.

**Table 1:** Characteristics of the study participants by SARS-CoV-2 variant type

	**Wild**	**Alpha**	**Delta**	**Omicron**			**Total**	***P*-value^a^**	***P* for trend^b^**

				**BA.1/BA.2**	**BA.5**	**Mixed**			
	**(n = 1,287)**	**(n = 1,720)**	**(n = 2,697)**	**(n = 21,823)**	**(n = 39,518)**	**(n = 9,999)**	**(n = 77,044)**		
Age									
18–64	993 (77.2)	1,376 (80.0)	2,516 (93.3)	18,445 (84.5)	32,339 (81.8)	NA	55,669 (72.3)	<0.001	NA
65–79	191 (14.8)	242 (14.1)	138 (5.1)	2,192 (10.0)	4,709 (11.9)	5,822 (58.2)	13,294 (17.3)		
80–109	103 (8.0)	102 (5.9)	43 (1.6)	1,186 (5.4)	2,470 (6.3)	4,177 (41.8)	8,081 (10.5)		
Gender									
Men	719 (55.9)	891 (51.8)	1,448 (53.7)	10,047 (46.0)	18,387 (46.5)	4,185 (41.9)	35,677 (46.3)	<0.001	<0.001
Women	568 (44.1)	829 (48.2)	1,249 (46.3)	11,776 (54.0)	21,131 (53.5)	5,814 (58.1)	41,367 (53.7)		
Vaccination status									
Unvaccinated	1,287 (100.0)	1,692 (98.4)	2,163 (80.2)	3,392 (15.5)	5,437 (13.8)	585 (5.9)	14,556 (18.9)	<0.001	NA
Dose 1	0 (0.0)	21 (1.2)	267 (9.9)	136 (0.6)	264 (0.7)	46 (0.5)	734 (1.0)		
Dose 2	0 (0.0)	7 (0.4)	264 (9.8)	14,658 (67.2)	9,408 (23.8)	363 (3.6)	24,700 (32.1)		
Dose 3	0 (0.0)	0 (0.0)	3 (0.1)	3,633 (16.6)	20,810 (52.7)	1,122 (11.2)	25,568 (33.2)		
Dose 4	0 (0.0)	0 (0.0)	0 (0.0)	4 (0.0)	3,599 (9.1)	4,610 (46.1)	8,213 (10.7)		
Dose 5	0 (0.0)	0 (0.0)	0 (0.0)	0 (0.0)	0 (0.0)	3,273 (32.7)	3,273 (4.2)		
Severe COVID-19									
Absent	1,250 (97.1)	1,637 (95.2)	2,649 (98.2)	21,713 (99.5)	39,435 (99.8)	9,840 (98.4)	76,524 (99.3)	<0.001	<0.001
Present	37 (2.9)	83 (4.8)	48 (1.8)	110 (0.5)	83 (0.2)	159 (1.6)	520 (0.7)		

Data are given as n (%). NA, not applicable. ^a^Chi-squared test. ^b^Cochran-Armitage test.

Data comparisons of proportions between groups based on the chi-squared test and the Cochran-Armitage test were made for the period from the wild type to the Omicron BA.5, because the Omicron Mixed period did not include patients under the age of 64.

**Fig. 2:**
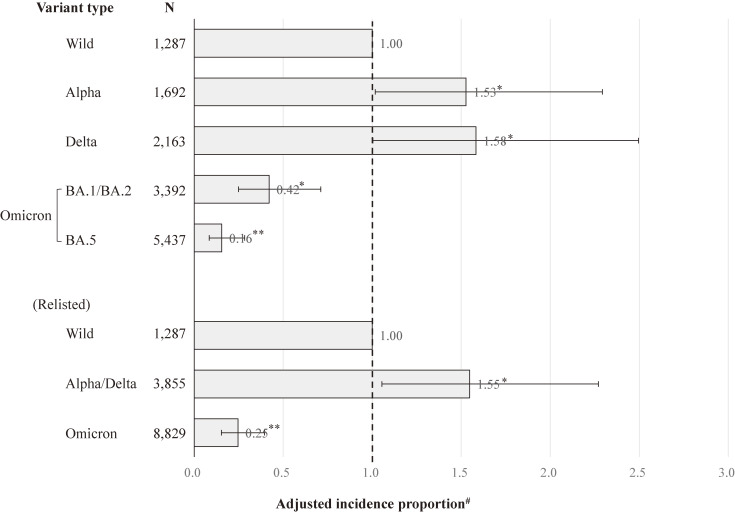
Association between SARS-CoV-2 variant type and severe COVID-19 compared with the wild-type among unvaccinated patients. **P < 0.001, *P < 0.05. Error bars display 95% confidence intervals. The Omicron Mixed was excluded from this analysis because it did not include those aged 64 years or younger. ^#^Adjusted for gender, age, the number of risk factors for aggravation, and the number of general hospital beds per population.

Regarding the association between SARS-CoV-2 variant type and severe COVID-19 compared with the wild-type among the unvaccinated patients (Fig. 2), the risk of severe COVID-19 exhibited a similar pattern for the Alpha variant and the Delta variant, and similar results were observed throughout the Omicron variant period. Therefore, we performed an analysis that reclassified the SARS-CoV-2 variant type into groups of the wild-type, the Alpha/Delta variants, and the Omicron variant. Among the unvaccinated patients, the AIP (95% CI) for severe COVID-19 was 1.55 (1.06–2.27) in the Alpha/Delta variants and 0.25 (0.15–0.40) in the Omicron variant, compared to the wild-type. After stratified analyses by age (Table 2), among people aged 18–64, the risk for severe COVID-19 relative to the wild-type significantly increased for the Alpha/Delta variants (AIP = 3.00, 95% CI = 1.37–6.57) and significantly decreased for the Omicron variant (AIP = 0.17, 95% CI = 0.06–0.46). Among people aged 65–79, the risk for severe COVID-19 relative to the wild-type showed no significant difference in the Alpha/Delta variants (AIP = 0.71, 95% CI = 0.37–1.39) and had a significant reduction during the Omicron variant (AIP = 0.22, 95% CI = 0.11–0.44). Among people aged 80–109, the risk for severe COVID-19 relative to the wild-type significantly increased for the Alpha/Delta variants (AIP = 2.00, 95% CI = 1.02–3.92), but not associated with the Omicron variant (AIP = 0.59, 95% CI = 0.27–1.29).

**Table 2:** AIP for severe COVID-19 relative to the wild type by age group among unvaccinated patients

**SARS-CoV-2** **variant type**	**Aged 18–64 (n = 12,631)**				**Aged 65–79 (n = 1,231)**				**Aged 80–109 (n = 694)**			
	**No. at risk**	**No. of cases**	**CuI** **(%)**	**AIP (95% CI)**	**No. at risk**	**No. of cases**	**CuI** **(%)**	**AIP (95% CI)**	**No. at risk**	**No. of cases**	**CuI** **(%)**	**AIP (95% CI)**
**Variant type**												
Wild	993	7	0.7	1.00	191	18	9.4	1.00	103	12	11.7	1.00
Alpha	1,359	36	2.6	3.18 (1.41–7.19)^*^	234	23	9.8	0.83 (0.42–1.62)	99	23	23.2	1.93 (0.97–3.83)^†^
Delta	2,092	37	1.8	2.86 (1.27–6.47)^*^	62	1	1.6	0.17 (0.02–1.29)^†^	9	4	44.4	2.61 (1.07–6.36)^*^
Omicron BA.1/BA.2	3,168	6	0.2	0.36 (0.12–1.05)^†^	135	10	7.4	0.61 (0.29–1.30)	89	8	9.0	0.68 (0.27–1.75)
Omicron BA.5	5,019	2	0.04	0.06 (0.01–0.33)^*^	266	4	1.5	0.12 (0.04–0.37)^**^	152	11	7.2	0.50 (0.21–1.23)
Omicron Mixed				--^§^	343	7	2.0	0.14 (0.05–0.38)^**^	242	22	9.1	0.62 (0.27–1.46)
**Relisted**												
Wild	993	7	0.7	1.00	191	18	9.4	1.00	103	12	11.7	1.00
Alpha/Delta	3,451	73	2.1	3.00 (1.37–6.57)^*^	296	24	8.1	0.71 (0.37–1.39)	108	27	25.0	2.00 (1.02–3.92)^*^
Omicron	8,187	8	0.1	0.17 (0.06–0.46)^**^	744	21	2.8	0.22 (0.11–0.44)^**^	483	41	8.5	0.59 (0.27–1.29)

AIP, adjusted incidence proportion; CI, confidence interval; CuI, Cumulative incidence. ^**^P < 0.001, ^*^P < 0.05, ^†^*P* < 0.10.

AIP was adjusted for gender, age, the number of risk factors for aggravation, and the number of general hospital beds per population in the municipality where the patient resided.

^§^Not estimated due to lack of available data.

**Fig. 3:**
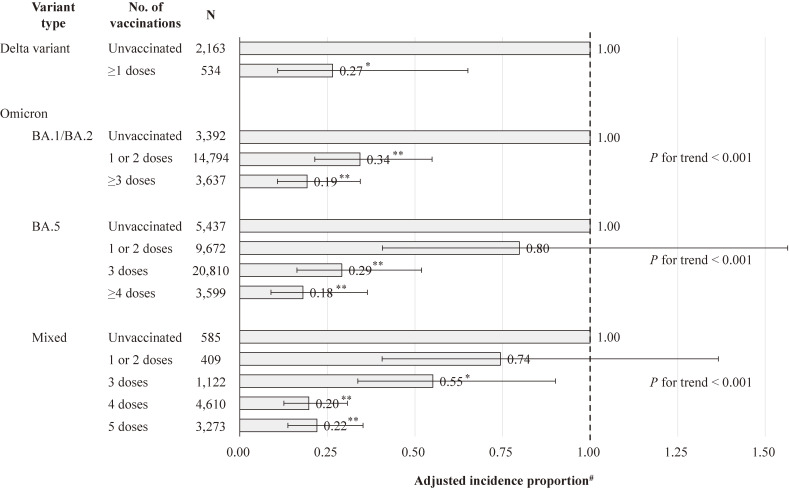
Association between no. of vaccinations and severe COVID-19 by variant type among all study participants. **P < 0.001, *P < 0.05. Error bars display 95% confidence intervals. The Omicron Mixed included only patients aged 65 and older. ^#^Adjusted for gender, age, the number of risk factors for aggravation, and the number of general hospital beds per population.

Regarding the association between the number of vaccinations and severe COVID-19 by variant type among all study participants (Fig. 3), vaccinations were significantly associated with prevention of severe COVID-19 for all variant types. In the Omicron variant, there was a dose-response relationship: the more vaccinations, the lower the risk of severe COVID-19 (*P* for trend <0.001). After stratified analyses by variant type and age (Table 3), in those aged 18–64, vaccination status had a significant association in the Delta variant and the Omicron BA1/BA.2, but no association in the Omicron BA.5. In those aged 65–79, a dose-response relationship was observed throughout the Omicron variant period (*P* for trend <0.001 in all types). In those aged 80–109, vaccination status had a significant association with prevention of severe COVID-19, regardless of variant type.

**Table 3:** Association of the number of vaccinations with severe COVID-19 by variant type and age group

**SARS-CoV-2** **variant type**	**No. of vaccinations**	**Aged 18–64**				**Aged 65–79**				**Aged 80–109**			
		**No. at ** **risk**	**No. of ** **cases**	**CuI** **(%)**	**AIP (95% CI)**	**No. at ** **risk**	**No. of cases**	**CuI** **(%)**	**AIP (95% CI)**	**No. at ** **risk**	**No. of cases**	**CuI** **(%)**	**AIP (95% CI)**
**Delta**	Unvaccinated	2,092	37	1.8	1.00	62	1	1.6	1.00	9	4	44.4	1.00
	1 or 2 doses	424	4	0.9	0.33 (0.12–0.89)^*^	76	0	0.0	NC	34	2	5.9	0.16^⁋^ (0.03–0.71)^*^
**Omicron**													
BA.1/BA.2	Unvaccinated	3,168	6	0.19	1.00	135	10	7.4	1.00	89	8	8.9	1.00
	1 or 2 doses	12,778	8	0.06	0.23 (0.08–0.66)^*^	1,405	19	1.4	0.17 (0.08–0.36)^**^	611	37	6.0	0.64 (0.30–1.34)
	≥3 doses	2,499	1	0.04	0.09 (0.01–0.92)^*^	652	6	0.9	0.11 (0.04–0.28)^**^	486	15	3.1	0.32 (0.14–0.75)^*^
				*P* for trend = 0.010				*P* for trend <0.001				*P* for trend = 0.003	
BA.5	Unvaccinated	5,019	2	0.04	1.00	266	4	1.5	1.00	152	11	7.6	1.00
	1 or 2 doses	9,347	8	0.09	2.23 (0.47–10.52)	191	1	0.5	0.37 (0.04–3.16)	134	5	3.6	0.57 (0.21–1.57)
	3 doses	17,092	6	0.04	0.57 (0.10–3.08)	2,566	11	0.4	0.31 (0.09–1.01)^†^	1,152	19	1.6	0.25 (0.12–0.51)^**^
	≥4 doses	881	1	0.11	1.07 (0.09–12.37)	1,686	2	0.1	0.07 (0.01–0.38)^*^	1,032	13	1.3	0.18 (0.08–0.40)^**^
				*P* for trend = 0.179				*P* for trend <0.001				*P* for trend <0.001	
Mixed	Dose 0					343	7	2.0	1.00	242	22	9.1	1.00
	1 or 2 doses					239	8	3.3	1.66 (0.62–4.43)	170	7	4.1	0.46 (0.20–1.04)^†^
	3 doses					709	8	1.1	0.61 (0.22–1.65)	413	22	5.3	0.57 (0.32–0.99)^*^
	4 doses					2,686	10	0.4	0.18 (0.07–0.47)^*^	1,924	38	2.0	0.21 (0.12–0.34)^**^
	5 doses					1,845	9	0.5	0.22 (0.09–0.59)^*^	1,428	28	2.0	0.22 (0.13–0.37)^**^
								*P* for trend <0.001				*P* for trend <0.001	

AIP, adjusted incidence proportion; CI, confidence interval; CuI, Cumulative incidence; NC, not calculated. ^**^P < 0.001, ^*^P < 0.05, ^†^*P* < 0.10.

AIP was adjusted for gender, age, the number of risk factors for aggravation, and the number of general hospital beds per population in the municipality where the patient resided. ^⁋^Because all cases were men and the number of general hospital beds per population had a no case category, gender and the number of general hospital beds per population were not included as covariates.

## Discussion

Using data from unvaccinated COVID-19 patients aged 18 and older, we found that the risk of severe COVID-19 was significantly lower in the Omicron variant period than in the wild-type period. However, among unvaccinated patients aged 80 and older, we could not observe any risk reduction in severe COVID-19 in the Omicron variant period. To the best of our knowledge, this is the first epidemiological study to assess the risk of severe COVID-19 during the entire period of the COVID-19 pandemic, based on community-based data. Furthermore, our results showed that vaccination was more effective in preventing severe COVID-19 in those aged 80 and older than in younger people. Therefore, this study demonstrates that vaccination is necessary and effective for people aged 80 and older who are at greatest risk of severe COVID-19.

Regarding comparison with previous studies, a community-based matched cohort study [14] reported a 64% increased risk of death in patients infected with the Alpha variant compared with those infected with the wild-type, and a retrospective cohort study [15] reported that the Omicron BA.1 variant had a 66% lower risk of death than the Delta variant. In addition, in animal experiments using hamsters, the Delta variant was highly fusogenic and more pathogenic than the wild-type [16], and the Omicron variant had reduced pulmonary infectivity and low pathogenicity compared to the Delta variant and the wild-type [17]. The attenuation of pathogenicity of the Omicron variant has been attributed to a low cleavage rate of the spike (S) protein and a reduced ability to destroy alveolar epithelial cells [18]. Summarizing these previous studies, compared to the wild-type, the risk of severe outcomes was higher in the Alpha and Delta variants and lower in the Omicron variant, which is consistent with our results among all study participants.

Age is a prime determinant of developing severe COVID-19, because it correlates with an increased proportion of people with major COVID-19 comorbidities, immunosenescence, endothelial damage, and coagulation dysfunction [19]. A large national cohort study [20] assessed age-specific estimates of the risk of severe outcomes for Omicron relative to Delta, and reported that the adjusted hazard ratio of death was approximately three times higher for unvaccinated patients aged at least 80 years compared with those aged 30–39. A recent study [21] reported that older people tended to have lower frequencies of specific memory B cell responses after the primary vaccination series than younger people, but that the booster vaccination produced a great increase in the frequencies of memory B cells for older people with low frequencies after the primary series. These previous studies [19–21] support our findings that among older people aged 80 and older, it is more difficult to reduce the risk of severe COVID-19, but that booster vaccination is more effective, compared to younger people.

We discuss the effects of potential bias that should be considered in this study. First, during the study period, testing methods for diagnosing COVID-19 were developed, and the ease of testing has changed. At the beginning of the pandemic, because the diagnostic method for COVID-19 was limited to the PCR test, it was not possible to test everyone who wanted it. Therefore, high-risk people (for example, older individuals, people with risk factors for severe disease, and people with severe symptoms at the time of diagnosis) were prioritized for testing. In Japan, antigen tests have been available since May 2020, and a COVID-19 testing system has been in place, especially since simple qualitative antigen test kits have become available. These improvements in testing capacity for COVID-19 have made it easier to diagnose and notify infected people, and there is a possibility that the proportion of patients with mild symptoms has increased. This may have contributed to the risk of severe COVID-19 being underestimated over time. On the other hand, because older people aged 80 and older were given priority testing even in the early stages of the pandemic, over time, people with mild symptoms did not become more prevalent among COVID-19 patients. This may be one reason why the reduction in the risk of severe COVID-19 was not observed during the Omicron mutation period in people over 80 years of age. Second, this study used surveillance data called the HER-SYS. During the Omicron variant period, the rapid increase in the number of infected people may have made it difficult to enter follow-up health outcomes into the HER-SYS, leading to an increase in underreporting of severe COVID-19 cases than during the period of other variants. This may have led to an underestimation of the risk of severe COVID-19 caused by the Omicron variant. Third, the vulnerable were more likely to die from COVID-19 in the earlier stages of the pandemic, while the healthy could survive and be vaccinated. That is, our findings may be influenced by an immortal time bias [22]. This bias has led to overestimating the effectiveness of vaccination in preventing severe COVID-19.

This study has several strengths. First, we used data from all COVID-19 patients notified to a public health center, and assessed the risk of severe COVID-19 from the wild-type to the Omicron variant. Second, we were able to consider age, gender, and risk factors for aggravation including comorbidities, smoking, obesity, and pregnancy, which are important confounding factors for the severity of COVID-19. Third, by limiting the analysis to non-vaccinated patients, we were able to assess the risk of severe COVID-19, which is not affected by the preventive effect of the vaccine. Although numerous studies have evaluated the risk of severe outcomes in COVID-19 patients [6, 7, 10, 13–15, 20, 23, 24], no studies have evaluated the risk of severe COVID-19 in non-vaccinated individuals throughout the pandemic period from the wild-type to Omicron variant.

This study has some limitations. First, people whose vaccination status was unknown had more severe illness than those whose vaccination status was identified (Additional file 1). If many of persons with unknown vaccination status had not been vaccinated and developed severe COVID-19, our results may have been underestimated. Second, in assessing the effectiveness of vaccines, it is necessary to consider previous infection status [20]. In this study, patients who were submitted as a first-time notification were considered to be patients with no previous infection and included in the analysis. However, this concept cannot be applied if the patient moved during the study period. For people moving during the COVID-19 pandemic, there is a possibility that they had already been infected, even if a first-time notification had been submitted. Third, during the Omicron variant period, in Japan, oral therapeutic drugs were as a rule used for all people aged 65 and older. Therefore, among older people aged 65 and older, the reduction in the risk of severe COVID-19 during the Omicron variant period may be attributed not only to the reduction in pathogenicity, but also to the effect of oral treatment. Finally, our findings are based on the data of all patients within the jurisdiction of one public health center in Nara Prefecture, and caution is required in generalizing our results.

## Conclusions

Among unvaccinated patients, the risk of severe COVID-19 was reduced in the Omicron variant compared to the wild-type, with significant variation with age. Especially in patients aged 80 and older, there was no significant difference in the risk of severe COVID-19 between the wild-type and the Omicron variant, and a cautious attitude is necessary regarding the reduction in pathogenicity. Regarding the preventive effect of vaccines against severe COVID-19, among patients aged 80 and older, more doses of vaccine were significantly associated with lower risk of severe COVID-19 for all variant types. In summary, our data show that older people aged 80 and older had less reduction in risk of severe COVID-19 during the Omicron variant duration, and a greater preventive effect of vaccines against severe COVID-19, compared to younger people. Our results highlight the importance of booster vaccinations for older people aged 80 and older.

## Abbreviations

AIP: adjusted incidence proportion
CI: confidence interval
COVID-19: Coronavirus disease 2019
HER-SYS: Health Center Real-time Information-sharing System on COVID-19
SARS-CoV-2: severe acute respiratory syndrome coronavirus

## Supplementary information

**Additional file 1.** Comparison of cumulative incidence of severe COVID-19 between persons with identified and unknown vaccination status. **Additional file 2.** Trend in the number of daily new COVID-19 cases in the study area. **Additional file 3.** Trends in the changing proportions of the lineage of SARS-CoV-2 in Nara, Japan. **Additional file 4.** Characteristics of the study participants by age group.

## Declarations

### Availability of data and materials

The data analyzed in this study are not publicly available.
