# Supplementary material for: Risk of severe COVID-19 in unvaccinated patients during the period from wild-type to Omicron variant: real-world evidence from Japan
Source: Environ Health Prev Med. 2024 Mar 5;29:10. doi: 10.1265/ehpm.23-00274 (PMC10937246; doi:10.1265/ehpm.23-00274)
Supplement: Supplementary file 1 — Additional file 1. Comparison of cumulative incidence of severe COVID-19 between persons with identified and unknown vaccination status. Additional file 2. Trend in the number of daily new COVID-19 cases in the study area. Additional file 3. Trends in the changing proportions of the lineage of SARS-CoV-2 in Nara, Japan. Additional file 4. Characteristics of the study participants by age group. [file ehpm-29-010-s001.docx]

**Additional file 1.** Comparison of cumulative incidence of severe COVID-19 between persons with identified and unknown vaccination status

| Variant  type | Vaccination  status | Aged 65 and older | | |  | Aged 18–64 | | |
| --- | --- | --- | --- | --- | --- | --- | --- | --- |
|  |  | No.  at risk | CuI of  severe cases | *P*^a^ |  | No.  at risk | CuI of  severe cases | *P*^a^ |
| Delta | Identified | 181 | 4.4% | 1.000 |  | 2,516 | 1.6% | 0.665 |
|  | Unknown | 7 | 0.0% |  |  | 93 | 2.2% |  |
|  |  |  |  |  |  |  |  |  |
| Omicron BA.1/BA.2 | Identified | 3,378 | 2.8% | <0.001 |  | 18,445 | 0.08% | 0.0503 |
|  | Unknown | 409 | 10.3% |  |  | 913 | 0.33% |  |
|  |  |  |  |  |  |  |  |  |
| Omicron BA.5 | Identified | 7,179 | 0.92% | <0.001 |  | 32,339 | 0.05% | 0.155 |
|  | Unknown | 236 | 7.2% |  |  | 2,391 | 0.13% |  |
|  |  |  |  |  |  |  |  |  |
| Omicron Mixed | Identified | 9,999 | 1.6% | <0.001 |  | NA | | |
|  | Unknown | 438 | 8.4% |  |  |  |  |  |

CuI, Cumulative incidence; NA, not available.

^a^Differences between identified and unknown groups were analyzed using Fisher’s exact test.

**
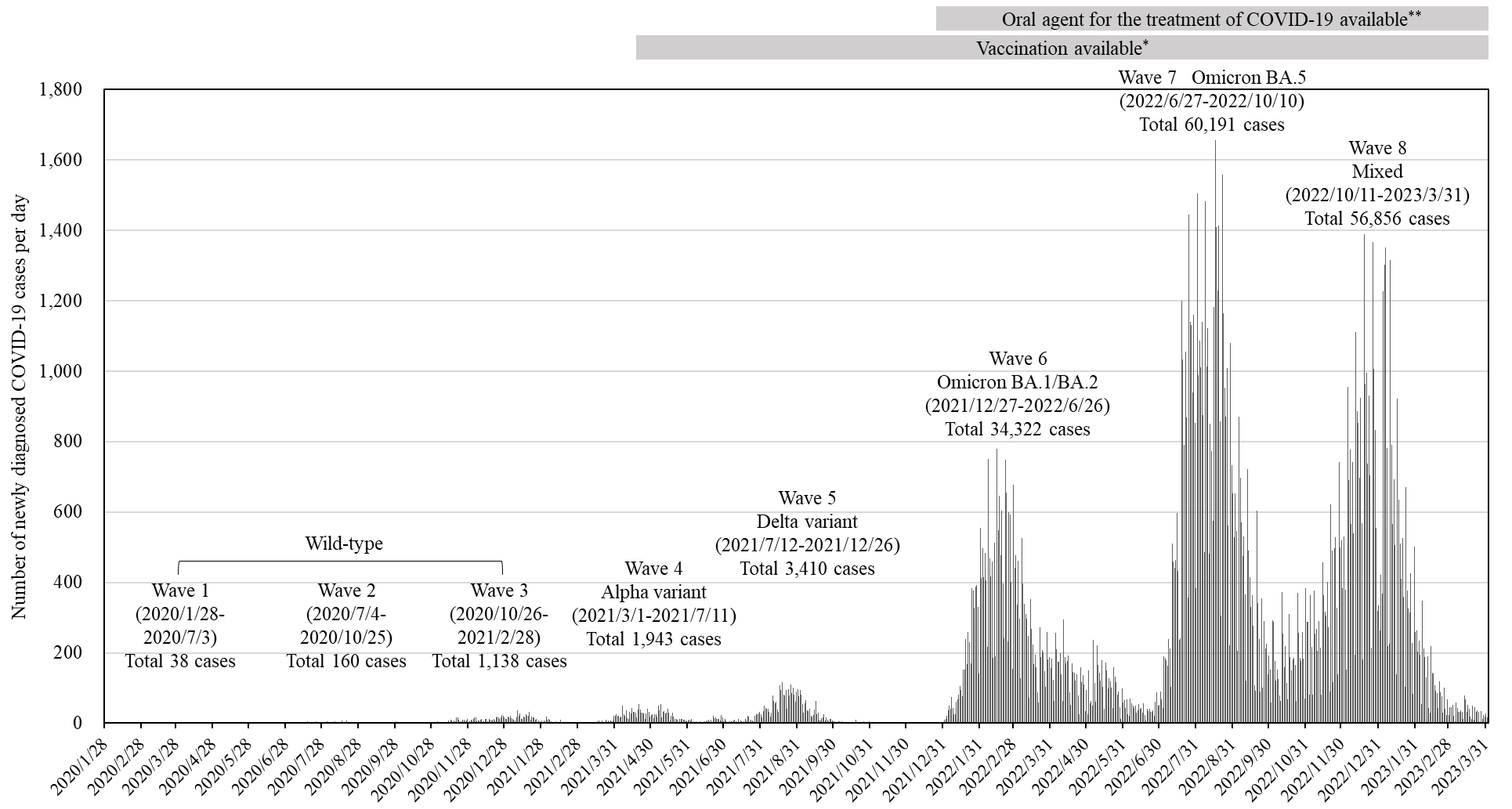
**

**Additional file 2.** Trend in the number of daily new COVID-19 cases in the study area. The study area is in the jurisdiction of Chuwa Public Health Center of the Nara Prefectural Government. ^*^Priority vaccination for older adults started on April 12, 2021. ^**^In Japan, on December 24, 2021, the anti-COVID-19 drug “Molnupiravir” (brand name, “Lagebrio”) received special approval and was prescribed to all people aged 65 or older.

**
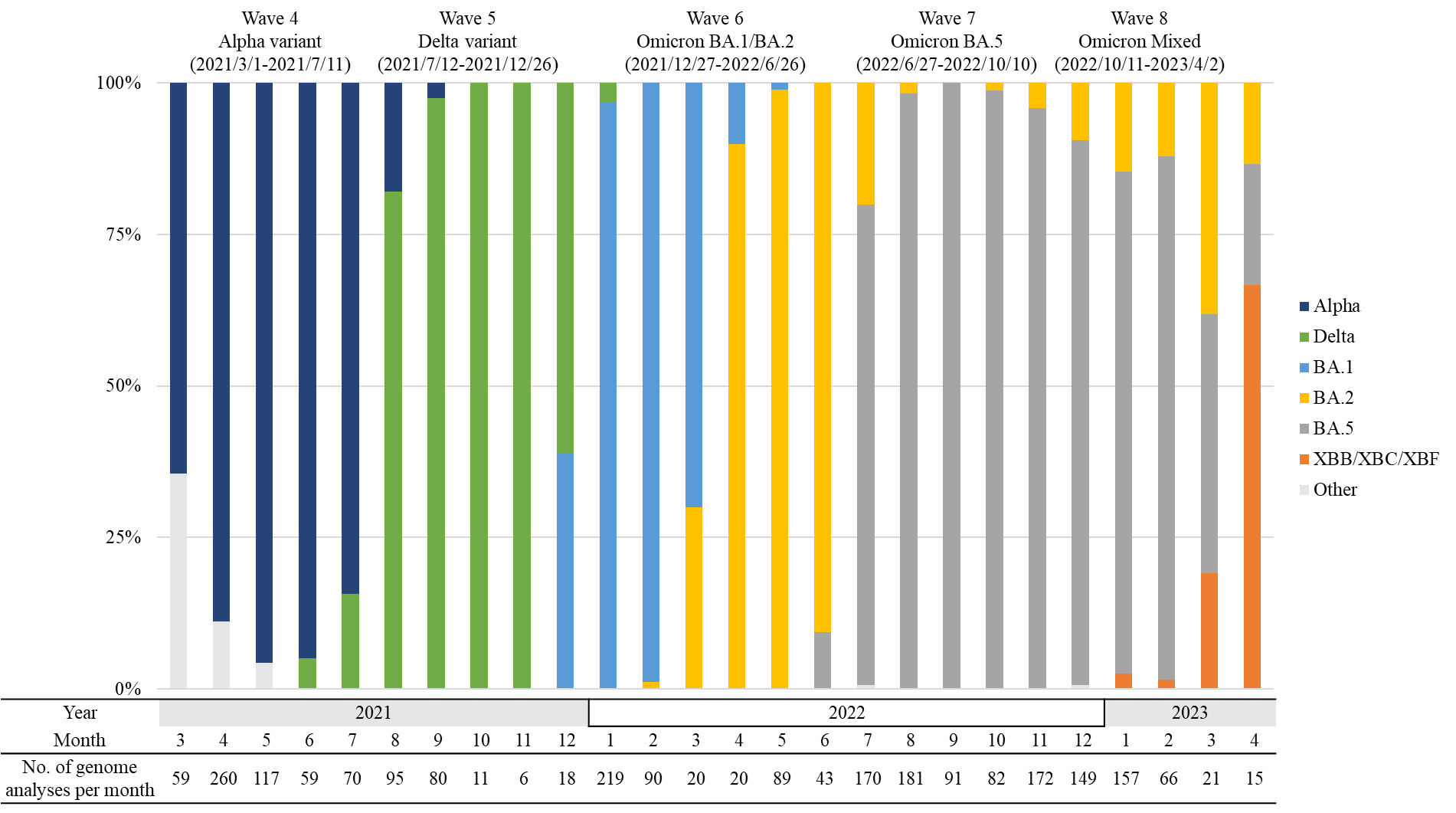
**

**Additional file 3.** Trends in the changing proportions of the lineage of SARS-CoV-2 in Nara, Japan. These data were provided by the Nara Prefectural Institute of Health. The genome analysis was conducted from March 2021 to April 2023. Because the genome analysis did not target all COVID-19 patients in Nara Prefecture, this data may not reflect the trend of Nara Prefecture as a whole.

**Additional file 4.** Characteristics of the study participants by age group

|  |  | Aged 18–64 | Aged 65–79 | Aged 80–109 | *P*-value^a^ | *P* for trend^b^ |
| --- | --- | --- | --- | --- | --- | --- |
|  |  | (n = 55,669) | (n = 13,294) | (n = 8,081) |  |  |
| Gender | |  |  |  |  |  |
|  | Women | 29,169 (52.4) | 6,949 (52.3) | 5,249 (65.0) | <0.001 | <0.001 |
| Aggravation risk factors | |  |  |  |  |  |
|  | Present | 13,005 (23.4) | 9,206 (69.2) | 6,488 (80.3) | <0.001 | <0.001 |
| No. of General hospital beds per 100,000 population | | | |  |  |  |
|  | <300 | 11,341 (20.4) | 2,837 (21.3) | 1,586 (19.6) | 0.007 | 0.810 |
| Vaccination status | |  |  |  |  |  |
|  | Unvaccinated | 12,631 (22.7) | 1,231 (9.3) | 694 (8.6) | <0.001 | <0.001 |
| Severe COVID-19 | |  |  |  |  |  |
|  | Present | 117 (0.2) | 137 (1.0) | 266 (3.3) | <0.001 | <0.001 |

Data are given as n (%). ^a^Chi-squared test. ^b^Cochran-Armitage test.
